# Anti-PD-1 immunotherapy with dose-adjusted ultra-hypofractionated re-irradiation in patients with locoregionally recurrent head and neck cancer

**DOI:** 10.1007/s12094-023-03172-y

**Published:** 2023-04-14

**Authors:** Ioannis M. Koukourakis, Axiotis G. Giakzidis, Michael I. Koukourakis

**Affiliations:** 1 Radiation Oncology Unit, 1st, Department of Radiology, Medical School, “Aretaieion” University Hospital, National and Kapodistrian University of Athens (NKUOA), Athens, Greece; 2 Department of Radiotherapy – Oncology, Medical School, Democritus University of Thrace, 68100 Alexandroupolis, Greece

**Keywords:** Head-neck cancer, Re-irradiation, Immunotherapy, Ultra-hypofractionation, PD-1

## Abstract

**Introduction:**

Patients with recurrent inoperable squamous-cell head-neck cancer (HNSCC) after chemo-radiotherapy have an ominous prognosis. Re-irradiation can be applied with some efficacy and high toxicity rates. Anti-PD-1 immunotherapy is effective in 25% of patients. Immunogenic death produced by large radiotherapy (RT) fractions may enhance immune response.

**Materials and methods:**

We evaluated the efficacy and tolerance of ultra-hypofractionated immuno-radiotherapy (uhypo-IRT) in 17 patients with recurrent HNSCC and 1 with melanoma. Four of HNSCC patients also had oligometastatic disease. Using a dose/time/toxicity-based algorithm, 7, 7 and 4 patients received 1, 2 and 3 fractions of 8 Gy to the tumor, respectively. Nivolumab anti-PD-1 immunotherapy was administered concurrently with RT and continued for 24 cycles, or until disease progression or manifestation of immune-related adverse events (irAEs).

**Results:**

Early and late RT toxicities were minimal. Three patients developed irAEs (16%). After the 12th cycle, 7/17 (41.2%) and 5/17 (29.4%) patients with HNSCC showed complete (CR) and partial response (PR), respectively. CR was also achieved in the melanoma patient. The objective response rates in HNSCC patients were 57%, 86% and 66%, after 1, 2 and 3 fractions, respectively (overall response rate 70.6%). Most responders experienced an increase in peripheral lymphocyte counts. The median time to progression was 10 months. The 3-year projected locoregional progression-free survival was 35%, while the 3-year disease-specific overall survival was 50%.

**Conclusions:**

Anti-PD1 uhypo-IRT is safe and effective in patients with recurrent HNSCC. The high objective response rates and the long survival without evidence of disease support further trials on uhypo-IRT.

## Introduction

Locoregional recurrence after chemo-radiotherapy for locally advanced squamous cell head-neck cancer (HNSCC) remains a significant risk following radical treatments for head and neck cancers [1], and is invariably morbid and distressing. Salvage is sometimes possible with surgery and/or radical re-irradiation but success rates are modest [2]. Re-irradiation with or without chemotherapy remains the only option with curative intent for the majority of patients with recurrent HNSCC. Retrospective studies show that the 2-year overall survival (OS) after re-irradiation ranges between 20–56% [3]. Severe toxicities occurring in about one-third of patients are, however, inevitable [4]. Conventional RT or hyperfractionation with low dose per fraction and localized fields is often applied in an attempt to keep radiation late sequel at acceptable levels. The dose of RT demanded to achieve acceptable locoregional control of the disease exceeds 60 Gy, which is high enough to induce fibrosis and necrosis of the already irradiated tissues.

Palliative cisplatin-based chemotherapy is another treatment option that mainly delays the fatal outcome of the disease, providing response rates between 10 and 40% [5], and a median survival of 10.1 months as reported in the EXTREME trial [6]. Palliative systemic treatment has been enhanced by the recent introduction of immunotherapies, albeit with responses seen only in a minority of patients. Anti-PD-1/PD-L1 immunotherapy has recently shown encouraging efficacy in metastatic and locally advanced HNSCC. The CheckMate 141 trial demonstrated an improvement of the median OS from 5.1 to 7.5 months compared to single-agent chemotherapy in patients with cisplatin-refractory disease [7]. In addition, the KEYNOTE-048 study showed that anti-PD1 pembrolizumab monotherapy improves median survival over chemotherapy when tumours express PD-L1 [8]. Tumour RT induces the interferon type I pathway and activates intratumoural dendritic cells, enhances the release of chemoattractant cytokines and modulates the microenvironmental conditions. These effects favor immune response, so that immunotherapy may be even more effective in irradiated tumours [9]. However, the low radiation dose per fraction used in conventional RT seems to be less effective in triggering the so-called radio-vaccination effect [10]. Thus, 1 to 3 large radiation fractions, as high as 8 Gy, in the range of sub-ablative and ultra-hypofractionated schemes, more efficiently induce the interferon type I response [11, 12].

Combination of immunotherapy with ultra-hypofractionated radiotherapy (ultra-hypofractionated immuno-radiotherapy; uhypo-IRT), using localized fields and doses expected to be tolerable, emerges as an appealing therapeutic hypothesis for patients with loco-regionally recurrent HNSCCs. Using the linear quadratic model and an *α*/*β*-ratio of 4 Gy for late responding tissues, one, two and three fractions of 8 Gy deliver a ‘biological equivalent to 2 Gy/fraction’ dose (EQD2) of 16, 32 and 48 Gy, respectively. As these dose levels are lower than the 60 Gy applied in standard re-irradiation, it is anticipated that uhypo-IRT may show low rates of radiation toxicities and high radio-vaccination effects. We investigated this hypothesis in the current pilot study. An algorithm to individualize the number of fractions of RT is created to minimize RT toxicities.

## Materials and methods

In 2019 we started treating with immuno-RT a wide range of carcinomas that recurred locally (without or with oligometastatic disease) in patients that had been treated with radical RT or chemo-RT. A combination of anti-PD-1 immunotherapy with palliative uhypo-RT (6-8 Gy doe per fraction) was applied.

Here, we present a retrospective analysis of 17 patients with recurrent inoperable (for surgical, medical or personal reasons) HNSCC after radical chemo-RT. Four of these patients had also oligomentastatic disease. One additional patient with melanoma was also included. As the RT dose applied ranged from 1 to 3 fractions of 8 Gy, the RT regimen falls into the palliative dose range. The choice of a number of fractions was left at the physicians’ discretion, as the number of fractions delivered to a previously irradiated area depends on time, location and existing radiation-induced toxicity. To minimize subjectivity and unify dose schedules, a consensus algorithm was created to guide the therapeutic decision for consultants of our department, as reported below. There was no dose escalation protocol as such an attempt was judged incompatible with the nature of disease and the necessity for dose level individualization according to the above mentioned and the reported parameters in the algorithm. As the expression of PD-L1 is not a prerequisite for anti-PD-1 immunotherapy in HNSCC, PD-L1 tumour study was not required for the recruitment of patients.

### Recruitment criteria

Eligible patients were aged above 18 years with locoregionally recurrent disease after CRT. Tumours were of epithelial origin or other histology arising in the pre-irradiated head-neck area. At least a 6-month interval since last RT fraction was demanded recruitment. Early recurrences were treated with chemotherapy aiming to accomplish at least 6 months since the last RT fraction before re-assessment for inclusion in the protocol. Responders to chemotherapy continued treatment until recurrence and then they were re-assessed for uhypo-IRT. Patients who relapsed after at least six months after chemo-RT were either unsuccessfully treated with cisplatin-based chemotherapy before recruitment in the protocol or were directly recruited in the immuno-RT in case of fragile patients with advanced age, ineligibility for cisplatin chemotherapy, or refusal to receive chemotherapy. Exclusion criteria were a PS higher than 1, documented grade 3 late radiation complications in the head-neck area targeted for re-irradiation, major heart, pulmonary, liver, kidney or psychiatric disease, active infection, autoimmune disease, HIV infection, organ transplantation, ongoing immunosuppressive therapy including corticosteroid treatment, and pregnancy. Patients with oligometastatic disease could be recruited in the trial, as all or major metastatic sites were to be treated with uhypo-RT.

### Pretreatment and treatment evaluation

Diagnosis of recurrent disease was based on CT-scan, MRI or PET-CT imaging, followed by a biopsy for pathological confirmation in cases with disputable clinical/imaging diagnosis. In cases where PET-scan was not performed, CT-scan of the chest and upper abdomen supplemented the head-neck imaging. Bone scan was performed only when patients reported relevant symptomatology, or when CT-scan or MRI suggested evidence of skeletal involvement. Full blood counts, glucose levels and biochemical kidney and liver function were assessed before recruitment. ECG was also performed. Thyroid function (TSH, T3 and T4), C-reactive protein (CRP) and creatine phosphokinase (CPK) levels were also assessed to obtain baseline values for monitoring immunotherapy-related adverse events (irAEs).

Response to uhypo-IRT was documented with CT- or MRI-scans performed after the 6th and the 12^th^ immunotherapy cycles, and every 4 months (or earlier if necessary) thereafter. The RECIST 1.1 criteria were used to define the response [13]. Complete response (CR) was documented after the elimination of the detectable disease. A remnant scar measuring < 5% of the initial dimensions was still considered as a CR. Decrease in the sum of the longest diameters (of all irradiated lesions) by more than 30% was considered as partial response (PR). Progressive disease (PgD) was defined as a > 20% increase in the longest dimension. All other cases were considered as stable disease (SD).

Immunotherapy and RT-related toxicities were recorded by consultation, clinical examination, hematological evaluation and standard biochemical tests before each cycle. The serum levels of CPK, C-reactive protein and thyroid function evaluation were recorded every three cycles. The NIH/NCI (National Institute of Health/National Cancer Institute) Common Terminology Criteria for Adverse Events (CTCAE) v 5.0 scale was used to score immunotherapy-related and acute radiation toxicity [14]. Regarding RT-toxicity, mucositis, fungal infections, skin toxicity and pain (or any other reported radiation-induced toxicity were scored before each RT fraction and monthly for the following two months. The LENT-SOMA toxicity scale was used to score late radiation sequel [15]. Post-irradiation fibrosis deterioration and tissue necrosis were the main concerns and were scored (together with any other reported toxicity) every 2 to 4 weeks during the immunotherapy period and every 4 to 6 months thereafter.

### Patient and disease characteristics

Table 1 shows the patient and disease characteristics. In 4 patients locoregional recurrence was accompanied by oligometastatic disease to the lungs (two patients with a single lung nodule), sternum (1 patient), or paratracheal space (1 patient). Seventeen tumours were of squamous cell histology. One patient with squamous cell laryngeal cancer pretreated with local and neck irradiation developed neck lymph node metastasis from a melanoma arising on the scalp (cytologically confirmed). In this case, re-irradiation concerned the involved neck area.

**Table 1:** Patient and disease characteristics

No pts	18
PS	
0	8
1	10
Gender	
Male	15
Female	3
Age	
Median	64.5
Range	46–86
Histology	
Squamous	17
Melanoma	1
Primary tumor location	
Oropharynx	4
Oral cavity	2
Larynx	3
Hypopharynx	3
Parotid	1
Lip	2
Auricular canal	1
Skin	2
Main recurrence area	
Oropharynx	3
Oral cavity	3
Larynx	2
Hypopharynx	1
Lip	1
Neck	8
Concurrent metastatic site	
Sternal mass	1
Paratracheal mass	1
Lung	2

### Radiotherapy details and dose algorithm

Table 2 shows the treatment characteristics. A volumetric modulated arc image-guided RT VMAT/IGRT technique was applied. A cone-beam CT was performed before each RT fraction. RT was applied only to the radiologically detectable disease. GTV comprised all detectable disease, and a margin of 0.5 cm was considered for CTV. A margin of 0.5 cm beyond CTV, followed by manual adjustment, was applied for PTV. Seven patients received 1 fraction of 8 Gy, seven received 2 weekly fractions of 8 Gy, and 4 patients 3 weekly fractions of 8 Gy. Organs at risk included the larynx, spinal cord, and tissue areas pre-irradiated at doses above 60 Gy. Metastatic lesions received 3 weekly fractions of 8 Gy using a VMAT/IGRT technique.

**Table 2:** Treatment characteristics

Previous therapy	
Radical chemo-radiotherapy	17
Postoperative chemo-radiotherapy	1
Time since first irradiation	
6–11 months	5
12–24 months	4
> 24 months	9
Fractionation	
8 Gy	18
Number of fractions	
1	7
2	7
3	4
Interfraction interval	
1 week	18
PTV margins beyond GTV	
1 cm	18
RT technique	
VMAT	18
Cone beam CT	
Before each fraction	18
RT for concurrent metastatic disease	
8 Gy/fraction	4
3 fractions	4

**Fig. 1:**
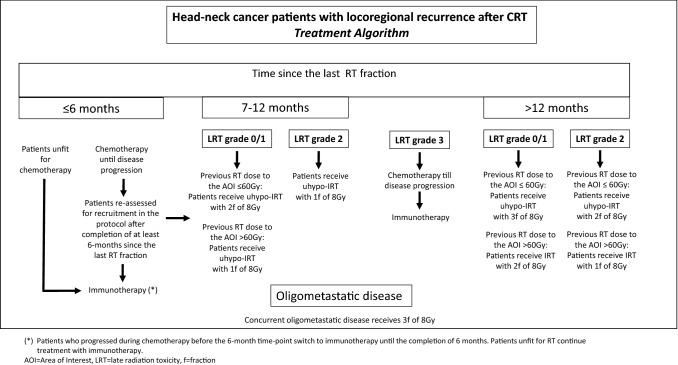
The algorithm created and used to guide the decision on the number of weekly 8 Gy fractions to treat patients with locoregional or oligometastic recurrent head-neck tumors (AOI = area of interest, LRT = late radiation toxicity, f = fraction)

The choice of the number of fractions delivered to patients was based on a created algorithm that takes into account: (i) the time since the last RT fraction (< 6, 6–12 or > 12 months), (ii) the grade of late radiation toxicity within the area considered for re-irradiation (grade 0/1, 2 and 3), and (iii) the total radiation dose delivered to the area considered for re-irradiation (< 60 Gy and > 60 Gy). Doses less than 60 Gy refer to the neck area, where the minimum dose was 50 Gy and the maximum to radiologically involved nodes 60 Gy. Doses higher than 60 Gy refer to the primary tumor area (range 64–70 Gy) or to areas of large neck lymph nodes (range 62–66 Gy). Late radiation toxicity (LRT) was scored before the onset of therapy according to the LENT-SOMA tables for muscle and soft tissues [15]. Briefly, limited palpable fibrosis with mild symptomatology was considered as grade 2, while extensive fibrosis with symptomatology was considered as grade 3. Figure 1 shows the treatment algorithm.

### Immunotherapy and dose-intensity de-escalation

Nivolumab anti-PD-1 immunotherapy was delivered at a dose of 240 mg iv., every two weeks, starting on the same day of the first fraction of RT. Immunotherapy was interrupted immediately after documentation of disease progression or after the development of any irAE, excluding controllable thyroid dysfunction [16]. For patients that did not reach CR, treatment continued until disease progression, every 2 weeks. For complete responders, a dose-intensity de-escalation algorithm for nivolumab administration was applied, as follows: nivolumab (same dose) was delivered every 3 weeks after the 12th cycle (6-month time point), and every 4 weeks after the 18th cycle (10.5-month time point) for a total of 24 cycles (16.5 months total treatment time).

### Endpoints- statistical analysis

The endpoints of the current study were RT tolerance, evaluation of immune-related toxicities, overall response rates, and 24-month locoregional progression-free survival (LPFS). LPFS and disease-specific OS were calculated with Kaplan–Meier curves, using the GraphPad Prism version 7.0 statistical package. The endpoints for LPFS and OS analysis were radiological, clinical or histopathological documentation of local or regional relapse, and death from cancer-related reasons, respectively.

Moreover, we assessed the impact of the lymphocyte counts before IRT and after 6 cycles of immunotherapy on the response rates. The non-parametric Kruskal–Wallis test with the subsequent Dunn test for intergroup comparison for multiple variables was used to compare groups of continuous tumor variables. A *p *value < 0.05 was considered for significance.

## Results

### Radiotherapy tolerance

Algorithm-guided RT, whether given in 1, 2 or 3 fractions, had an excellent tolerance. Mucositis grade 2 appeared in 2/4 cases receiving 3 fractions. None of the patients developed errhysis or hemorrhage. No other early toxicity was noted. At the time of the last follow-up, there was no late toxicity recorded for patients receiving 1 or 2 fractions. One out of 4 patients receiving 3 fractions to the neck area complained of neck pain without any signs of aggravation of local fibrosis. The pain regressed within 3 months after irradiation.

### Immunotherapy related toxicities

At the time of analysis, a median of 14 immunotherapy cycles had been administered (range 3–24). Out of 18 patients, 3 (17%) developed irAEs. One patient developed grade 3 hepatitis after the 3rd cycle. The second patient, presented with a grade 3 maculo-papular skin rash after the 6th cycle. The third patient complained about severe asthenia, accompanied by a conspicuous increase in serum CPK levels, compatible with rhabdomyolysis, after the 12th cycle. All three patients interrupted immunotherapy permanently.

### Response

**Fig. 2:**
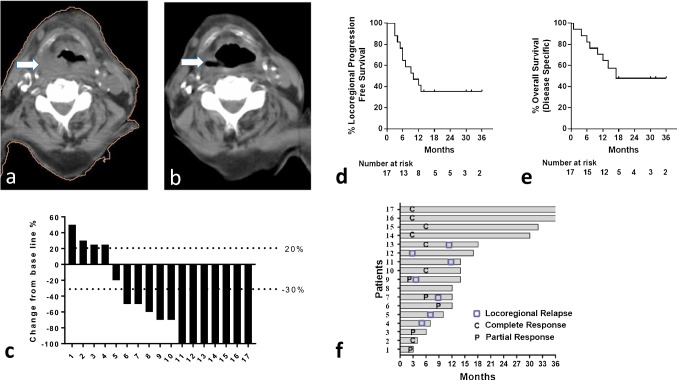
Response and survival figures. **a**, **b** CT-images of a recurrent tumor in the supra-laryngeal area (**a**) that achieved CR after uHypo-IRT (**b**) (white arrows show the tumor area); **c** waterfall plot of maximum reductions of tumor size; **d**, **e** Kaplan–Meier locoregional progression-free survival (**d**) and disease-specific overall survival (**e**) curves of patients with recurrent head-neck tumors treated with uhypo-IRT; **f** swimmer plot of patients showing the maximum follow-up, the time point when loco-regional relapse appeared, and the time points when complete and partial responses were documented

Response analysis focuses on cases with squamous histology, so we excluded the one patient with melanoma, who has accomplished 2-year of follow-up without disease progression. After the 6th immunotherapy cycle (3 months of therapy), 4/17 (23.5%) and 7/17 (41.2%) patients showed CR and PR, respectively, on CT-scan evaluation. One patient with PR, documented after the 3rd cycle, interrupted therapy due to irAE and died from PgD. This patient was included as a partial responder. Two patients had disease stabilization and 4/18 had PgD. After the 12th cycle (6 months of therapy), 7/17 (41.2%) and 5/17 (29.4%) patients displayed CR and PR, respectively. Table 3 shows the distribution of responses according to the number of RT fractions. The objective response rates (after the 12 cycle) were 57%, 86% and 66% in patients receiving 1, 2 and 3 fractions, respectively. The overall response rate was 70.6%. Three out of 4 irradiated metastatic lesions achieved CR and did not further progress. Figure 2a, b presents a typical case of a supra-laryngeal recurrent mass with CR, while Fig. 2c highlights the waterfall plots of maximum response rates in the 17 patients.

**Table 3:** Distribution of tumor response in 17 patients with squamous cell cancer according to the number of radiotherapy fractions

	No of patients	
	6th cycle	12th cycle
1 fraction		
CR	2/7 (28.6%)	3/7 (42.8%)
PR	2/7 (28.6%)	1/7 (14.3%)
SD	1/7 (14.3%)	1/7 (14.3%)
PgD	2/7 (28.6%)	2/7 (28.6%)
2 fractions		
CR	2/7 (28.6%)	2/7 (28.6%)
PR	3/7 (42.8%)	4/7 (57.1%)
SD	1/7 (14.3%)	–
PgD	1/7 (14.3%)	1/7 (14.3%)
3 fractions		
*Primary/neck recurrence*		
CR	0/3 (0%)	2/3 (66%)
PR	2/3 (66%)	–
SD	–	–
PgD	1/3 (33%)	1/3 (33%)
3 fractions		
*Metastatic lesions*		
CR	3/4 (75%)	3/4 (75%)
PR	–	–
SD	–	–
PgD	1/4 (25%)	1/4 (25%)

CR, complete response; PR, partial response; SD, stable disease; PgD, progressive disease

### Lymphocytes and response

**Fig. 3:**
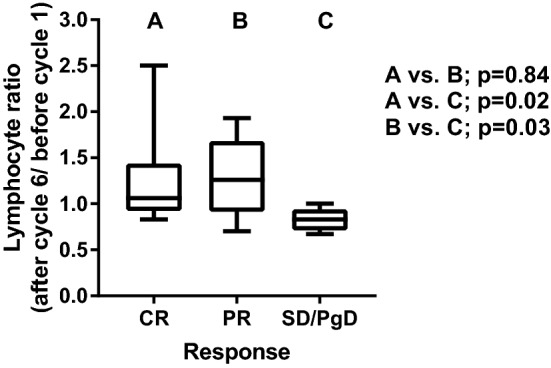
Lymphocyte ratios (after cycle 6/before cycle 1) according to the response rates obtained after uhypo-IRT (CR = complete response, PR = partial response, SD/PgD = stable disease/progressive disease)

The pre-IRT lymphocyte counts had no impact on the response rates. The lymphocyte ratio between the counts after the 6th cycle and before the onset of immunotherapy was calculated. This ranged from 0.6 to 2.5, median 1.1. A significant association was noted. Patients who failed to show an objective response had a significantly lower lymphocyte ratio compared to complete and partial responders (*p* = 0.02 and *p* = 0.03, respectively). Most responders experienced an increase in lymphocyte counts, while the inverse was noted in non-responders; Fig. 3.

### Dose de-escalation, disease progression and irAE

Out of 8 complete responders who entered the de-escalation phase of immunotherapy, 1 progressed locally at 13 months and 2 died from the intercurrent disease at 14 and 18 months, while all others are alive and free of progression. Three of them have completed the 24th cycle. Two complete responders interrupted immunotherapy due to irAE after the 6th and 12th cycle, and are free of disease 24 and 36 months after the onset of therapy. Both irAEs occurred during the first phase of immunotherapy.

Out of 5 partial responders, who were planned to continue the bi-weekly immunotherapy until disease progression, one interrupted therapy after the 3rd cycle due to irAE and had progressive disease shortly thereafter. Four patients had disease progression, after the 8th, 12th, 17th and 20th cycle, respectively.

### Survival analysis

Survival analysis comprised patients with squamous cell histology. The median time of follow-up of all patients was 13 months (range 1–36 months). Eleven out of 17 patients progressed locoregionally during immunotherapy. The median time to progression was 10 months. The 2 and 3-year projected LPFS was 35%. The Kaplan–Meier LPFS analysis is shown in Fig. 2d.

Eight patients died from locoregional disease progression; three of them with concurrent distant metastasis. Three patients died due to intercurrent diseases without cancer progression. The median OS is undefined as yet. The projected 3-year OS was 50%. The Kaplan–Meier disease-specific survival analysis is shown in Fig. 2e.

Figure 2f shows the Swimmer plot of patients presenting the maximum follow-up, the time point when loco-regional relapse appeared, and the time points when complete and partial responses were documented.

## Discussion

Radiotherapy is gradually emerging as a potent tool to enhance the efficacy of modern immunotherapy with immune checkpoint inhibitors. Indeed, experimental studies show that RT enhances HLA-class-I antigen presentation and induces dendritic cell activation by triggering the interferon type 1 response pathway in cancer cells and tumour infiltrating dendritic cells [17]. At the clinical level, however, the combination of RT with anti-PD-L1 immunotherapy for the radical treatment of locally advanced HNSCC failed to show a benefit in the JAVELIN trial [18]. Moreover, the GORTEC 2015-01 trial testing the combination of RT with anti-PD-1 immunotherapy vs. cetuximab did not result in a survival benefit, although immunotherapy was linked with better tolerance [19].

The aforementioned studies were conducted with conventionally fractionated RT, which may be the cause of the failure to exploit the radio-vaccination effect induced by RT. The fractionation and dose of RT that produces the optimal radio-vaccination demanded to sensitize the tumours to immunotherapy is under investigation [20]. It seems that repeated large fractions of 6-8 Gy of radiation are more appropriate than standard 2 Gy/fraction schedules or ablative high-dose RT [21]. Schaue et al. showed in in vivo models that two fractions of 7.5 Gy can increase the anti-tumour immune response in the spleen, better than other lower or higher fractions [22]. In a study by Vanpouille et al., even one fraction of 8 Gy strongly produced high levels of double-stranded DNA fragments and induced the expression of IFNAR1 in cancer cells [12].

Taking into account the available experimental data [23], it is suggested that 3 fractions of 8 Gy is an optimal schedule to induce the interferon type I response and enhance the efficacy of immunotherapy, although a lower number of fractions, even one, has important activity. The biological equivalent dose (EQD2) provided by such schedules to normal late-responding tissues (*α*/*β* = 4 Gy) is estimated to 16, 32 and 48 Gy, for 1, 2 and 3 fractions, respectively. These doses are expected to have excellent tolerance in terms of late radiation complications in previously untreated patients. Early toxicity is also expected to be low, as the EQD2 for early responding tissues (*α*/*β* = 10 Gy) is estimated to 12, 24 and 36 Gy for 1, 2 and 3 fractions, respectively. The fact that the linear quadratic model supports a good tolerance of hypofractionated 8 Gy/fraction RT, combined with the experimental evidence that 8 Gy fractions are optimal for radio-vaccination, strongly support trials of uhypo-IRT.

Another reason why the two immuno-RT clinical trials failed to show a survival benefit may rely on the irradiation of the tumour-draining lymph nodes (TDLNs), which is the standard approach of radical RT for HNSCC. As TDLNs are the central station for the activation of cytotoxic T-cells by primed dendritic cells arriving from the irradiated tumour through the lymphatics, daily neck irradiation may kill T-cells and block the radio-vaccination effect [24]. Recurrent tumours after RT provide an excellent model to test localized hypofractionated RT with limited or even no neck irradiation as a means for radio-vaccination and enhancement of the activity of immune checkpoint inhibitors.

In the current trial, we tested the above hypothesis in a subgroup of head and neck cancer patients with ominous prognosis, those with recurrent inoperable disease after previous CRT. Re-irradiation is considered a standard approach, but the dose demanded to anticipate acceptable efficacy exceeds 60 Gy, which is inevitably linked with high rates of severe fibrosis and necrosis, often with fatal outcome [4]. Using a dose/time/toxicity algorithm, we treated three cohorts of patients with 1, 2 and 3 fractions of 8 Gy together with nivolumab anti-PD1 immunotherapy. As predicted by the linear quadratic model the relative low EQD2 administered had minimal early toxicity without any late radiation sequel. Immunotherapy showed also an excellent tolerance with irAE recorded in 3/18 patients, who interrupted immunotherapy. This excellent tolerance profile justifies large-scale evaluation of uhypo-IRT.

The above suggestion was further justified by the high overall response rates of 70.6%, where 41.2% concerned CR. Objective responses of 57% were noted even after the administration of only one RT fraction, and these were above 80% in patients receiving 2–3 fractions. This high efficacy of uhypo-IRT was followed by a projected 3-year LPFS of 35%, and a disease-specific OS of 50%. Looking into the results of re-irradiation in IMRT studies that delivered a median radiation dose of 60 Gy, the reported 2-year locoregional control ranges from 38–65% and the 2-year OS from 40–58% [3]. The results of the current study compare favorably to the published experience with conventional CRT, considering the low radiation dose administered to the patients. The results are far better than the expected from chemotherapy alone, where the expected 2-year survival is lower than 30% [6].

In a previously reported phase II randomized trial by McBride et al., on 62 patients with metastatic HNSCC, nivolumab immunotherapy was administered together with SBRT (3 fractions of 9 Gy) to one selected metastatic lesion [25]. The study focused on the abscopal effects of RT, anticipating increased immunotherapy-achievable response rates in non-irradiated lesions. Despite the negative results reported, other randomized studies in non-small cell lung cancer have substantiated an abscopal effect from hypofractionated RT [26]. In contrast to the aforementioned trial in HNSCC, our study did not attempt to substantiate abscopal, but rather ‘in-field’ beneficial effects from the combination of uhypoRT with immunotherapy. Thus, we investigated whether the ‘in situ’ radio-vaccination effect potentiates the local activity of immunotherapy. Whether, the abscopal effect, thus reduction of metastatic disease progression could have been also achieved is difficult to conclude from the current small study.

Another observation from the current study was that 7/8 patients achieving CR with uhypo-IRT have not relapsed, despite the dose-intensity de-escalation schedule of immunotherapy applied after the first 6 months and the fact that 2 of them interrupted prematurely immunotherapy due to irAEs. The way time and dose-intensity factors affect the maintenance of CR after immunotherapy remains unclear. Even minimal exposure to immunotherapy has been suggested to be critical for tumour eradication in certain groups of patients [27], which could potentially apply to tumours receiving uhypo-IRT. The CheckMate 153 trial concerning advanced non-small cell lung cancer demonstrated that nivolumab immunotherapy beyond 1 year was associated with additional benefit; this finding, however, was based on a mixed group of patients with CR and PR [28]. Although preliminary, the data of the current study support the idea that immunotherapy dose-intensity de-escalation with immunotherapy protraction at 16.5 months (instead of a fixed 12-month immunotherapy) sustains its anti-tumour efficacy without producing further irAEs.

As the expression of PD-L1 is not a prerequisite for anti-PD-1 nivolumab immunotherapy in HNSCC, PD-L1 tumor study was not required for the recruitment of patients. The efficacy of nivolumab in HNSCC has been independent of the PD-L1 status in the trials that led to its approval. Nevertheless, PD-L1 is an inducible gene, and RT is well known to up-regulate PD-L1 in experimental studies [29, 30]. The role of PD-L1 in IRT, whether before or after irradiation is obscure. In this study, we could not assess its prognostic value, as pre-RT PD-L1 was available in only 7 cases (making any analysis unreliable), and post-RT biopsy was not included in the treatment protocol. Analysis of lymphocyte ratios (after IRT/before IRT), however, provided an interesting association, suggesting that low lymphocyte counts after 6 cycles of immunotherapy (3 months since the onset of therapy) predicts poor response to therapy. These results are in accordance with a study on solid tumors by Diehl et al., where baseline or persistent lymphopenia during immunotherapy was linked with poor progression-free survival [31]. In a study by Ho et al., on 34 HNSCC patients treated with ICIs, low pre-treatment lymphocyte counts were linked with poor response to therapy [32].

It is concluded that uhypo-IRT with anti-PD1 immunotherapy is a promising therapeutic approach for patients with recurrent after chemo-radiotherapy head and neck cancer, who are ineligible for salvage surgery. The favorable objective response rates of 80% in patients receiving at least 2 fractions of 8 Gy, and the long survival without evidence of disease reaching a projected 3-year OS of 50%, support further clinical evaluation of uhypo-IRT as a therapeutic option for patients with locoregionally recurrent head-neck cancer.

## Data Availability

All data reported in the study are available in the files of patients kept in our Department.
